# Auxotrophy-based curation improves the consensus genome-scale metabolic model of yeast

**DOI:** 10.1016/j.synbio.2024.07.006

**Published:** 2024-07-30

**Authors:** Siyu Han, Ke Wu, Yonghong Wang, Feiran Li, Yu Chen

**Affiliations:** a State Key Laboratory of Bioreactor Engineering, East China University of Science and Technology, Shanghai, 200237, China; b Key Laboratory of Quantitative Synthetic Biology, Shenzhen Institute of Synthetic Biology, Shenzhen Institute of Advanced Technology, Chinese Academy of Sciences, Shenzhen, 518055, China; c Institute of Biopharmaceutical and Health Engineering, Tsinghua Shenzhen International Graduate School, Tsinghua University, Shenzhen, 518055, China

**Keywords:** *Saccharomyces cerevisiae*, Genome-scale metabolic model, Auxotrophy, Flux balance analysis

## Abstract

*Saccharomyces cerevisiae*, a widely utilized model organism, has seen continuous updates to its genome-scale metabolic model (GEM) to enhance the prediction performance for metabolic engineering and systems biology. This study presents an auxotrophy-based curation of the yeast GEM, enabling facile upgrades to yeast GEMs in future endeavors. We illustrated that the curation bolstered the predictive capability of the yeast GEM particularly in predicting auxotrophs without compromising accuracy in other simulations, and thus could be an effective manner for GEM refinement. Last, we leveraged the curated yeast GEM to systematically predict auxotrophs, thereby furnishing a valuable reference for the design of nutrient-dependent cell factories and synthetic yeast consortia.

## 1 Introduction

Genome-scale metabolic models (GEMs) are usually constructed using bottom-up approaches [1], integrating genomic information with accumulated knowledge of an organism's metabolic capabilities to reconstruct a comprehensive map of metabolism [2]. The emergence of whole genome sequencing technology, coupled with the exponential advancements in computational power [3], has propelled the construction of a multitude of GEMs spanning an expansive diversity of phylogenetic realms [4] and plays a pivotal role in enhancing the performance of microbial cell factories [5,6].

To enhance the predictive power of these models, their continuous curation and refinement are essential [7]. There are several ways of curation in the field [8]: (ⅰ) curated growth phenotype predictions based on experimental data [9], (ⅱ) refined the model with detailed lipid metabolism, tRNA synthesis, and transport processes, leveraging information based on metabolic databases [10], (ⅲ) curated gene-reaction rules informed by gene essentiality data and synthetic lethality analyses. [11], (ⅳ) enhanced the predictive power of environment-specific models through the integration of omics data [12].

The model organism *Saccharomyces cerevisiae* has been widely used in the production of chemicals [13], biofuels [14] and recombinant proteins [15]. Its GEM is particularly important in metabolic engineering and systems biology [16]. Since the publication of the first GEM for *S*. *cerevisiae* in 2003 [17], there has been a rich history of model development [18,19]. In 2019, a new consensus metabolic network Yeast8 was published [20]. And so far, the GEM for yeast has been updated to Yeast9 [12]. While numerous methods already exist for curating metabolic models, we propose that leveraging auxotrophy data can further enhance the predictive performance of GEMs.

We curated a database for GEM using known auxotroph experiments in *S. cerevisiae*. Using this database, we performed auxotrophy-based curation on the yeast consensus GEM Yeast9. Subsequently, we compared the curated model with Yeast9 in predicting growth phenotypes. We demonstrated that this curation improved the predictive power of the GEM in terms of auxotrophy prediction and did not affect other simulations. Finally, all nutrient auxotrophs in the curated model were predicted, facilitating the design and optimization of yeast cell factories and consortia for advancing biotechnology and promoting sustainable bioproduction.

## 2 Materials and methods

### 2.1 Experimental data for curation

All data about auxotrophic experiments were downloaded from The *Saccharomyces* Genome Database (SGD, https://www.yeastgenome.org/). A database was compiled for model refinement after screening according to the information listed and the primary literature cited therein.

All unfiltered initial data can be found in the Supplementary Dataset 1.

The experimental data utilized for curation can be found in the Supplementary Dataset 2. The data were presented as gene-compound pairs, where the gene represented a knockout that results in inviability, and the compound represented the nutrient for which the strain with the gene knockout was auxotrophic i.e., addition of this compound rescued growth.

### 2.2 Models and tools

The model used for curation was based on the consensus GEM of *S*. *cerevisiae* Yeast9. In phenotype prediction, the previous version of the yeast model Yeast8 was also utilized.

Flux balance analysis (FBA) stands as a widely employed computational modeling technique in metabolic modeling, dedicated to elucidating and predicting cellular metabolism within the confines of constraint-based metabolic frameworks [21]. FBA allows for the prediction of genetic and environmental perturbations and thus serves as a suitable method to predict gene essentiality and nutrient utilization. Moreover, FBA has been adopted in these specific simulations during the curation of Yeast8 and Yeast9. Therefore, we also used FBA in our study to simulate auxotrophs.

FBA in this study was performed in MATLAB using the COBRA toolbox [22]. All optimizations were performed utilizing the Gurobi solver (https://www.gurobi.com/).

### 2.3 Model growth prediction

To test the consistency between the model predictions and experimental phenotypic data of auxotrophy, FBA was employed for model predictions. Growth simulation used maximization of the growth rate (i.e., rate of the biomass reaction) as an objective function. The predicted growth rate of the default model Yeast9 was 0.0859, and we set a threshold at 1 % of this growth rate. Any prediction falling below the threshold was classified as inviable. In the experiment, the mutant strain was inviable in the absence of a specific compound, yet it grew upon the introduction of this compound. The model simulation approached this in two stages.

1) The model simulated single-gene knockout using the function “deleteModelGenes” in the COBRA toolbox. FBA was utilized to predict whether the simulation was viable.
2) By employing the "changeRxnBounds" function, the lower flux boundaries for the exchange reactions related to specific compounds were set to −1000 mmol/gDw/h, which is the default minimum bound in the model and thus allows for unconstrained uptake to rescue the growth. Subsequently, FBA predictions were rerun to predict whether the simulation was viable.

### 2.4 Model curation

To guarantee the accuracy and high quality of the refined reactions and metabolites, detailed annotations from databases such as SGD [23], KEGG [24], Rhea [25], and UniProt [26] were meticulously integrated. Model curation primarily encompasses the following approaches.

1) Curate gene-reaction associations. This step involved examining the database to confirm the accuracy of associations between genes and reactions in the model. It further entailed investigating whether alternative compensatory genes existed that could potentially take over the metabolic role, thereby guaranteeing the precise annotation of all alleles implicated in these processes.
2) Block reaction. If the model was predicted to grow after gene knockout, this implied the compound could be synthesized without relying on the reaction associated with this gene. Using FBA, the fluxes of all reactions in the system were determined both before and after the gene knockout. Changes in these fluxes were examined to reveal other synthesis pathways. Subsequently, the biological plausibility of these pathways was validated using databases, and it was assessed whether deactivating the original reaction was feasible, with the model being refined accordingly.
3) Change reaction coefficients. To alter reaction stoichiometries, the correct coefficients first needed to be calculated. Taking chitin as an example, if it was intended to be incorporated into a carbohydrate pseudoreaction, the stoichiometric coefficients of the other compounds involved had to be proportionally adjusted. The procedure entailed initially converting the stoichiometric coefficients of all reactants into mass units, this conversion being was done by multiplying them with their respective molar masses. Once chitin was included, the new total mass was determined. To ret mass balance, each original reactant mass had to be scaled down, utilizing the ratio of the initial total mass to the final total mass (now inclusive of chitin). Lastly, these adjusted masses were divided by their respective molar masses to regain the revised coefficients for the modified reaction, this adjustment being a necessary part of the process.
4) Add reactions. In instances where contradictions arose between the synthesis reactions of compounds in the model and those in the database, leading to inaccurate growth predictions, the original synthesis reactions in the model were deactivated, and the correct reactions were incorporated instead. Furthermore, metabolites that yeast can synthesize de novo may not have been included in the model exchange reactions, despite their actual presence. Consequently, adding exchange reactions for such substances facilitated the successful simulation of their auxotrophy.

### 2.5 Model accuracy calculation

The model growth prediction capability has been previously introduced in Section 2.3. Here, prediction accuracy was quantified as the ratio of correctly predicted gene-compound pairs to the overall dataset size. Based on the criteria of predicting viability after gene knockouts, and the ability to predict growth recovery upon adding the corresponding compound after gene knockouts, the quantities of various scenarios were calculated both before and after the model curation.

### 2.6 Simulations on other phenotypes

Utilizing the “sub_use” function in Yeast-GEM multi-omics analysis (https://GitHub.com/hongzhonglu/yeast_GEM_multi_omics_analysis), a comparative analysis was conducted on the utilization of carbon, nitrogen, phosphorus, and sulfur sources between Yeast9 and Yeast9_curated models. Gene essentiality was determined using the “essentialGenes” function from yeast-GEM (https://GitHub.com/SysBioChalmers/yeast-GEM).

For screening gene-compound pairs, all genes and exchange reactions in the model were taken as inputs, replicating the procedures outlined in Section 2.3, which led to the identification of nutrient auxotrophs within the models.

### 2.7 Systematic auxotrophy prediction

First, we acquired all the genes, exchange reaction IDs, and names of exchange compounds in the model. A loop was constructed to individually knock out each gene and compute the growth rate using FBA for every scenario. Building upon this, a new loop was devised: for the gene that was knocked out, the exchange reactions of compounds were sequentially activated, followed by recalculating the growth rate for each case. The algorithm applied mirrored the two-step process outlined in Section 2.3. We documented the genes and compounds that met the criteria of being inviable in the first step and viable in the second step, ensuring that the obtained compounds were precisely matched to the respective genes through this method.

## 3 Results and discussion

### 3.1 Auxotrophy data screening

**Fig. 1:**
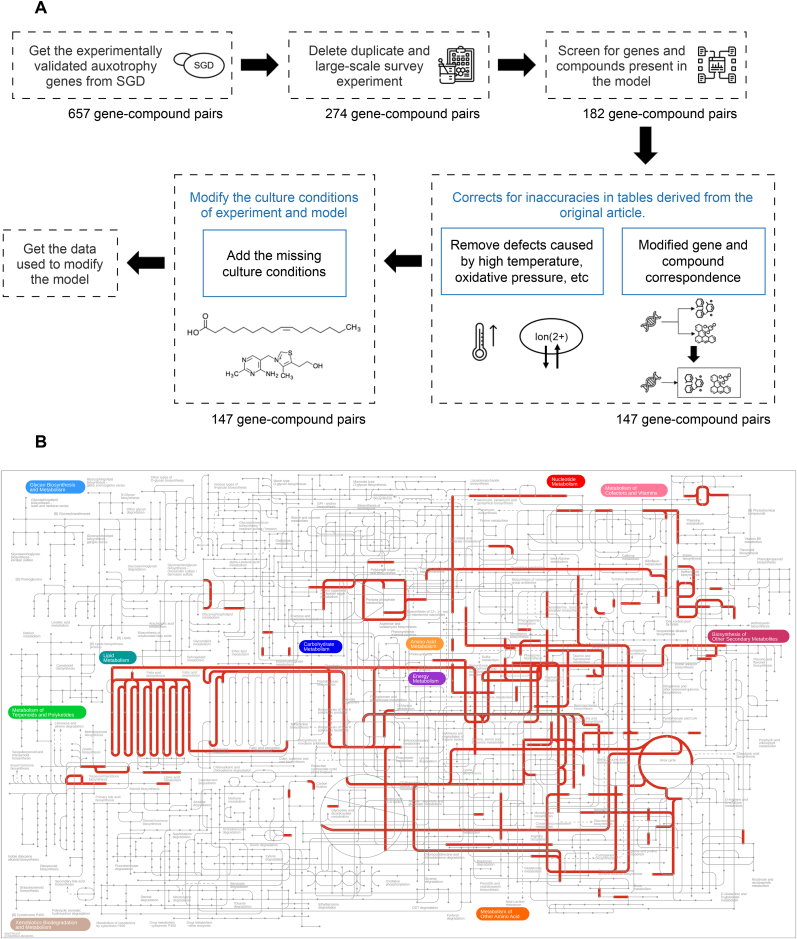
(A) A workflow for screening auxotrophy data in SGD. (B) Distribution of the genes included in the screened dataset. The genes are visualized as red lines on the metabolic map.

We downloaded the auxotrophy data for *S*. *cerevisiae* from SGD, comprising a total of 657 gene-compound pairs. The initial data contain information such as genes, experiment types, strain backgrounds, and chemicals (Supplementary Dataset 1). To screen for the usability of this dataset, we devised a flowchart (Fig. 1A).

First, based on the experiment type category, data from large-scale survey experiments [27] were excluded from our analysis. After this screening process, we obtained 274 gene-compound pairs.

Second, genes not in the Yeast9 model were also excluded from the curation process. For the compounds in the experiment, we attempted to match corresponding exchange reactions. However, specific compounds such as sphingoid [28], β-alanine [29], dTMP [30,31], and long-chain fatty acid were found to lack direct exchange reactions in the current model. Consequently, some of these compounds were excluded from the present analysis. Thus, 182 gene-compound pairs remained.

Third, the dataset obtained via direct download contains inaccuracies. Certain inconsistencies emerged when comparing the representation of data on the SGD against the original experimental reports. By carefully examining and cross-referencing with the primary literature, we have addressed these discrepancies, ensuring that our data reflects the most accurate and reliable information available. One type of inaccuracy is in the correspondence between genes and compounds. It is necessary to supplement with two (or more) substances after gene knockouts, resulting in simulation discrepancies [32,33]. A case is the requirement for mutants lacking *HOM2*, where growth is supported by homoserine or (methionine and threonine) [32]; however, the raw dataset incorrectly represents this as homoserine, methionine, or threonine independently. The other issue is that instances of auxotrophy attributed to other factors, such as high temperature [34] and oxidative stress [35], were not considered in the analysis. This step involved correcting and removing a small amount of data, resulting in 147 gene-compound pairs.

Finally, we observed that experimental conditions varied across studies. The use of nutrient-rich medium or Yeast Nitrogen Base (YNB) medium may ignore the auxotrophy of nutrients present in the medium [36]. For example, auxotrophy experiments were carried out under YNB medium conditions, where the deletion of *ADE4* allowed growth upon supplementation with adenine [37]. However, while the auxotrophic strain utilized adenine, it also utilized thiamine present in the YNB medium. Consequently, to align with experimental conditions, thiamine was included in the medium to simulate this specific case. This step did not involve any reduction of the gene-compound pairs, ultimately leading us to get the data containing 147 gene-compound pairs used to modify the model.

Meticulous screening is imperative for its application in the curation of GEMs. Our study furnishes a meticulously curated database that can serve as a foundation for curation based on auxotrophs.

The metabolic pathways of all the genes were obtained through screening using iPath 3 (https://pathways.embl.de/) based on KEGG annotation (Fig. 1B). The genes were primarily involved in key functional categories including amino acid transport and metabolism, nucleotide metabolism, cofactors and vitamins metabolism. A few were distributed in lipid metabolism, biosynthesis of other secondary metabolisms, carbohydrate metabolism, energy metabolism, terpenoids, and polyketide metabolism.

### 3.2 Model curation

We compared the model predictions with the experimental phenotypes by conducting two steps of growth simulations (Materials and methods). In the correctly predicted situations, where the predictions align with the experimental phenotypes, gene knockouts should lead to inviability and the addition of the appropriate compounds should then recover viability. The incorrect predictions by the model are twofold. Firstly, there are instances where a gene knockout does not result in the expected inviability. We designate such scenarios as type I. Secondly, there are cases where a gene knockout results in inviability, yet the subsequent introduction of the theoretically appropriate compound does not facilitate the resumption of growth. These incorrect predictions are classified as type II. Based on the incorrect predictions, we curated the model as documented in Table 1.

**Table 1:** The gene-compound pairs in the model along with the specific approaches employed for curation.

Gene-compound pairs	Error type	Reactions	Reaction equations	Curation	References
*AAT2*-aspartate	type I	r_0217	l-glutamate[m] + oxaloacetate[m] => 2-oxoglutarate[m] + l-aspartate[m]	block reaction	[38]
*ALD2* and *ALD3*-pantothenic acid	type II	r_0172	3-aminopropanal[c] + H2O[c] + NAD[c] => beta-alanine[c] + 2 H + [c] + NADH[c]	change the associated gene from “YMR110C or YMR169C or YMR170C″ to “YMR169C or YMR170C″	[39,40]
*CHO2*-choline	type I	r_2488 r_2489 r_2490 r_2491 r_2492 r_2493 r_2494 r_2495	phosphatidylethanolamine[erm] + S-adenosyl-l-methionine[erm] => H + [erm] + S-adenosyl-l-homocysteine[erm] + phosphatidyl-N-methylethanolamine[erm]	change the associated gene from “YGR157W or YJR073C″ to “YGR157W″	[41]
*CYS3*-cysteine	type I	r_0312	hydrogen sulfide[c] + O-acetyl-l-serine[c] => acetate[c] + l-cysteine[c]	block reaction	[[42], [43], [44]]
*CYS4*-cysteine		r_4703	TRX1[c] + 3-mercaptopyruvate[c] <=> hydrogen sulfide[c] + pyruvate[c] + TRX1 disulphide[c]	block reaction	
*ERG10*-ergosterol	type I	r_0559	acetoacetyl-CoA[c] + acetyl-CoA[c] + H2O[c] => 3-hydroxy-3-methylglutaryl-CoA[c] + coenzyme A[c] + H + [c]	block reaction	[45]
*GFA1*-d-Glucosamine	type I	r_4048	0.74851 (1->3)-beta-D-glucan[ce] + 0.25009 (1->6)-beta-D-glucan[ce] + 0.36141 glycogen[c] + 0.71094 mannan[c] + 0.13828 trehalose[c] => carbohydrate[c]	add chitin to the carbohydrate pseudoreaction with a coefficient of −0.02361, The coefficients of other reactants are adjusted proportionally.	[[46], [47], [48]]
		r_0477	d-fructose 6-phosphate[c] + l-glutamine[c] => alpha-d-glucosamine 6-phosphate[c] + l-glutamate[c]	change the associated gene from “YMR084W or YKL104C″ to “YKL104C″	
*GSH1-*glutathione	type I	r_4598	0.00019 coenzyme A[c] + 1e-05 FAD[c] + 0.00265 NAD[c] + 0.00015 NADH[c] + 0.00057 NADP(+)[c] + 0.0027 NADPH[c] + 0.00099 riboflavin[c] + 1.2e-06 TDP[c] + 6.34e-05 THF[c] + 1e-06 heme a[c] => cofactor[c]	add glutathione to cofactor with a coefficient of -1e-06	[49,50]
*ERG13*-ergosterol *ERG20*-ergosterol *HEM1*-heme *HEM12*-heme *HEM2*-heme *HEM3*-heme *HEM4*-heme	type II	r_temp1	heme a[e] =>	add heme a exchange reaction	[51,52]
*MET3*-methionine	type I	r_1026	ADP[c] + H + [c] + sulfphate[c] => 5′-adenylyl sulfate[c] + phosphate[c]	block reaction	[53]
*MET13*-methionine	type I	r_0080	5,10-methylenetetrahydrofolate[c] + H + [c] + NADPH[c] => 5-methyltetrahydrofolate[c] + NADP(+)[c]	change the associated gene from “YGL125W and YPL023C″ to “(YGL125W and YPL023C) or YGL125W″	[54,55]
*MET17*-methionine	type I	r_0815	l-cysteine[c] + O-succinyl-l-homoserine[c] <=> H + [c] + l-cystathionine[c] + succinate[c]	block reverse reaction	[56]
*HOM2*-methionine and threonine *HOM3*-methionine and threonine *HOM6*-methionine and threonine *MET2*-methionine *THI4*-thiamine	type II	r_temp2	ADP-5-ethyl-4-methylthiazole-2-carboxylate[c] + H2O[c] - > AMP[c] + 4-methyl-5-(2-phosphonooxyethyl)thiazole[c] + carbon dioxide[c] + H + [c]	add reaction HET-P synthase (thiazole synthase)	[[57], [58], [59]]
		r_temp3	l-glycine[c] + NAD[c] + hydrogen sulfide[c] - > nicotinamide[c] + ADP-5-ethyl-4-methylthiazole-2-carboxylate[c] + 3 H2O[c] + H + [c]	add reaction adenylated thiazole synthase	
		r_2070	H + [c] + l-cysteine[c] + l-glycine[c] + O-acetyl-l-homoserine[c] + ribose-5-phosphate[c] => 4-methyl-5-(2-phosphonooxyethyl)thiazole[c] + acetate[c] + ammonium[c] + carbon dioxide[c] + gamma-aminobutyrate[c] + 3 H2O[c] + pyruvate[c]	block reaction	
		r_2071	d-xylulose 5-phosphate[c] + H + [c] + l-cysteine[c] + l-glycine[c] + O-acetyl-l-homoserine[c] => 4-methyl-5-(2-phosphonooxyethyl)thiazole[c] + acetate[c] + ammonium[c] + carbon dioxide[c] + gamma-aminobutyrate[c] + 3 H2O[c] + pyruvate[c]	block reaction	
*URA2*-uracil	type II	r_0250	2 ATP[c] + bicarbonate[c] + H2O[c] + l-glutamine[c] => 2 ADP[c] + carbamoyl phosphate[c] + 2 H + [c] + l-glutamate[c] + phosphate[c]	change the associated gene from “YJL130C and YJR109C and YOR303W″ to “YJL130C or (YJR109C and YOR303W)”	[60]

#### 3.2.1 Curation of incorrect predictions of type I errors

This situation was accomplished by precisely controlling the metabolic pathways, ensuring accurate representation and functionality. For instance, upon *AAT2* knockout, the model predicted growth, conflicting with experimental observations [61]. By examining the metabolic flux distribution, we identified a compensatory alternative reaction r_0217 catalyzed by Aat1p, functioning in the mitochondria. While Aat1p could provide a backup, the cytosolic pathway was found to be the predominant source of overall aspartate aminotransferase activity [38]. Therefore, we blocked reaction r_0217 to ensure the correct prediction for the *AAT2*-aspartate pair.

Besides the simple cases, we found that the curation of the *THI4*-mediated thiamine synthesis pathway proved to be complex. Through the analysis of flux distribution, we discovered that two reactions, namely r_2070 and r_2071, generated intermediates within the thiamine biosynthesis pathway, even though they lacked both genetic correlations and supporting evidence from the literature [57,58]. Meanwhile, the gene *THI4* was not associated with the reaction in Yeast9 [62,63]. To address this discrepancy, we blocked the reactions r_2070 and r_2071 and assigned the gene *THI4* with the correct reaction [59] was integrated into the model. Moreover, blocking the reactions r_2070 and r_2071 also addressed the incorrect predictions related to the genes *HOM2*, *HOM3*, *HOM6* [32], and *MET2* [53]. These genes produced O-acetyl-l-homoserine, which acted as a substrate for reaction r_2070 and r_2071, consequently affecting the production of thiamine. These genes, involved in the methionine pathway, produced the intermediate metabolite O-acetyl-l-homoserine, which was incorrectly consumed as substrates by reactions r_2070 and r_2071, thereby affecting the biosynthesis of thiamine.

In addition to blocking bypasses, we also adjusted the biomass composition of the model. Gfa1p played a key role in d-glucosamine synthesis and ultimately the production of chitin [64]. In *S*. *cerevisiae*, chitin was mainly found in the primary septum, although a small amount appears to be dispersed throughout the cell wall [65,66]. As a critical structural constituent, chitin significantly contributed to the integrity and stability of fungal cell walls [67,68]. However, chitin was not added to the yeast GEMs [17], resulting in a zero flux through the chitin synthesis pathway. Thus, we incorporated chitin into the carbohydrate pseudoreaction and adjusted the composition ratio of carbohydrates [46,47].

#### 3.2.2 Curation of incorrect predictions of type II errors

This situation was primarily addressed by correcting the gene-reaction associations. For example, *MET13* played a crucial role in methionine biosynthesis in *S*. *cerevisiae* [53]. Upon knockout of *MET13*, the mutant became auxotrophic and required exogenous supplementation of methionine for growth. In Yeast9, the gene encoding methylenetetrahydrofolate reductase (MTHFR), which was involved in folate metabolism closely related to methionine synthesis, was identified as YGL125W (*MET13*) or YPL023C (*MET12*). However, the individual disruption of *MET12* did not affect growth whereas the individual disruption of *MET13* led to methionine auxotrophy, and the deletion of both *MET12* and *MET13* also resulted in a reliance on exogenous methionine for growth [54,55]. Furthermore, attempts to compensate for the *MET13* deletion by overexpressing *MET12* did not alleviate the methionine auxotrophic condition [54]. Accordingly, we modified the associated genes of reaction r_0080 from YGL125W or YPL023C to (YGL125W and YPL023C) or YGL125W, which led to the correct prediction for the *MET13*-methionine pair.

In summary, we curated 12 gene-reaction associations, blocked 8 reactions, and introduced two novel synthesis reactions and one additional exchange reaction. Moreover, we adjusted the coefficients of the cofactor and carbohydrate pseudoreactions.

### 3.3 Performance of GEM after curation

**Fig. 2:**
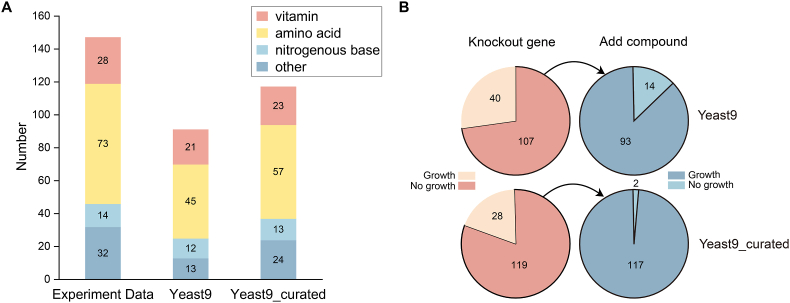
Performance of GEM. (A) Number and classification of gene-compound corrections. (B) The result of simulations after gene knockout and supplement of the corresponding compound. All displayed genes are experimentally essential.

To evaluate the curation, we predicted the growth of auxotrophic strains using both Yeast9 and its curated version, i.e., Yeast9_curated, and subsequently calculated the percentage of predictions that correctly matched the gene-compound pairs within the database. Compared to Yeast9, our curation escalated the prediction accuracy from 63.27 % to 79.59 % (Fig. 2A).

Based on the primary metabolic pathways of genes and the types of paired compounds, we categorized the gene-compound pairs into four groups: amino acids, vitamins, purine and pyrimidine bases, and others (Fig. 2A). The latter category, labeled as “others”, predominantly includes compounds like polyamines [29,69] and sterols [70,71], which have been extensively studied in their respective biosynthetic pathways. Our curation focused on amino acids and other unclassified compounds. We found that Yeast9_curated correctly predicted more gene-compound pairs in the amino acids and other groups than Yeast9.

Auxotrophy data indicated that for gene-compound pairs, the initial prediction was inviability upon gene knockout (i.e., gene essentiality), with subsequent restoration of viability anticipated upon compound supplementation. We employed the models to predict for both Yeast9 and Yeast9_curated, yielding the predicted outcomes post-gene deletion and compound addition (Fig. 2B). Compared to Yeast9, Yeast9_curated increased the number of gene essentiality from 107 to 119 and elevated the number of correct predictions from 93 to 117 while decreasing the number of incorrect predictions. Our curation process thereby enhanced the performance in predicting gene essentiality and the validity of auxotrophic phenotype predictions.

**Fig. 3:**
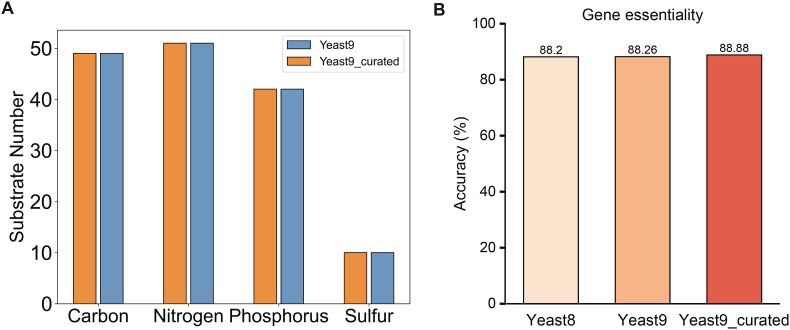
Simulations of (A) substrate usage and (B) gene essentiality.

We performed substrate utilization predictions by Yeast9_curated and found that the results were comparable with those by Yeast9 (Fig. 3A). To evaluate the computational efficiency of the models before and after curation, we employed the substrate utilization prediction experiment in Fig. 3A as a benchmark, and we found the time required was 6m24.8s for Yeast9 and 6m32.8s for Yeast9_curated. It can be considered that the curation would not largely affect the computational efficiency. We also independently corrected the GEM using data from *S. cerevisiae* S288c and evaluated its performance (Fig. S1). Information regarding the strain backgrounds for all gene-compound pairs is mentioned in the Supplementary Dataset 2.

In addition, we performed gene essentiality prediction using Yeast8, Yeast9, and Yeast9_curated. There was an enhancement (i.e., 0.62 %) in the accuracy of predicting gene essentiality after auxotrophy-based curation, which was even much greater than that (i.e., 0.06 %) when progressing from Yeast8 to Yeast9 (Fig. 3B). Therefore, the auxotrophy-based curation did not decrease the model performance in predicting substrate utilization and gene essentiality.

### 3.4 Systematic auxotrophy prediction by the curated GEM

**Fig. 4:**
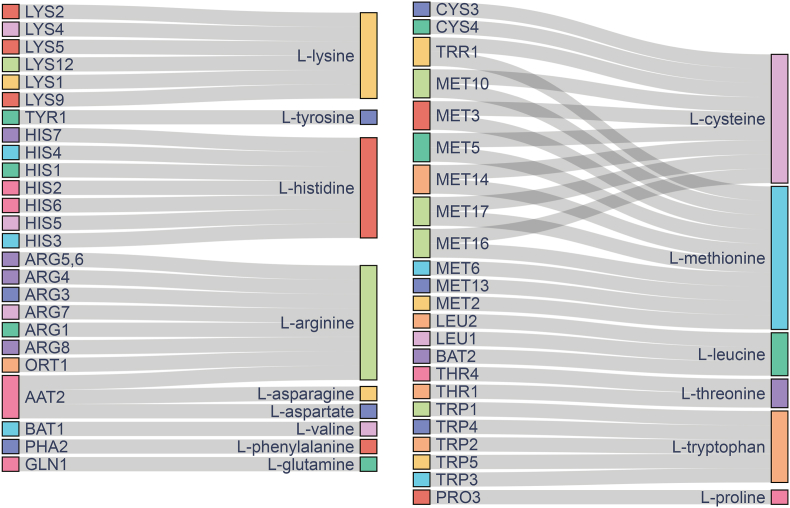
Prediction of essential genes and their corresponding auxotrophs related to amino acid metabolism. The genes represent the deficient genes in auxotrophic strains, and the compounds connected to them denote the nutrients required for these strains.

With Yeast9_curated, we systematically predicted auxotrophy by deleting essential genes and then supplementing amino acids. We found that the outcomes (Fig. 4) generally covered the data compiled in SGD, while also providing experimentally unidentified gene-compound pairs. By harnessing the comprehensive information derived from our analyses, we can determine which compounds need to be added post-gene knockout to restore growth, serving as a valuable reference for designing synthetic yeast communities [72].

The simulations showed that while most amino acid biosynthesis pathways did not overlap with one another, cysteine and methionine displayed a close relationship as they shared a common precursor, homocysteine [73]. Cysteine and methionine could interconvert with homocysteine, thereby implying that supplementing with either one could restore the growth of the mutant strains with an impaired biosynthesis pathway [74].

In addition, the simulations did not predict that the addition of serine, isoleucine, alanine, glycine, and glutamic acid could restore the growth of mutant strains. While knockout of *SER1* or *SER2* could lead to serine auxotrophy [75], model simulations showed that serine could theoretically be produced through the reverse reactions of r_0502 and r_0503, which convert glycine into serine. We hypothesized that metabolic regulations or inhibitory factors under the experimental conditions might suppress these reverse reactions in practice, thereby preventing serine synthesis. Due to the lack of these specific constraints or regulatory mechanisms, the model failed to reflect this inhibition. The situation for isoleucine is similar to that for serine [76]. Alanine, glycine, and glutamic acid feature multiple synthesis pathways and lack dedicated synthesis genes, rendering their prediction more intricate.

Moreover, we conducted comprehensive gene knockouts with supplementation of all metabolites that have exchange reactions in the model (Fig. S2). We found that the supplementation with S-adenosyl-l-methionine rescued the greatest number of knockout mutants. This intervention bridges the methionine and adenosine metabolic pathways, encompassing a series of intermediates. Additionally, the biosynthetic pathways for vitamins such as riboflavin and nicotinate are predominantly correlated with growth, further highlighting their essentiality in cellular metabolism.

### 3.5 Limitations of the current model framework

There are some incorrect predictions that could not be addressed properly. To facilitate future model improvement, we have presented two scenarios: (ⅰ) metabolism of enzyme cofactors and (ⅱ) prediction of intermediate metabolite fluxes. These would inspire new avenues of exploration and refinement for upcoming model iterations.

**Fig. 5:**
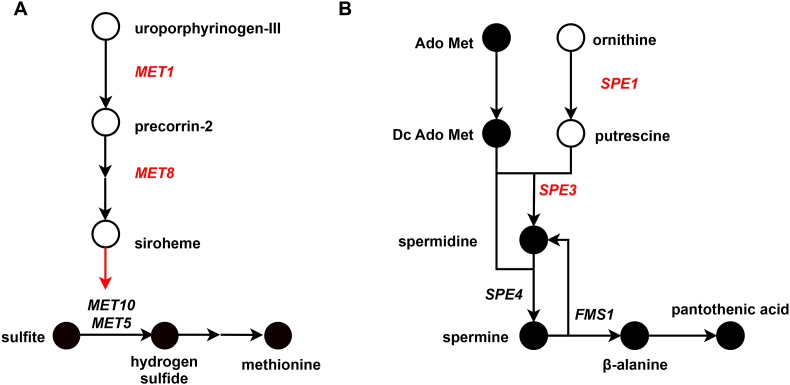
The related synthesis pathways of (A) siroheme and (B) spermine and spermidine. In the FBA simulation with maximization of growth, there are metabolites with non-zero flux (solid circles) and metabolites with zero flux (open circles). Red genes: predicted non-essential genes, implying the model still predicts growth upon their deletion. Red pathways: pathways not adequately implemented in the model.

For the first scenario, one example was related to uroporphyrinogen III, a pivotal intermediate in siroheme biosynthesis [[77], [78], [79]]. In yeast, the consumption of uroporphyrinogen III was catalyzed by Met1p, while dehydrogenase and chelatase activities of *MET8* are required for the following two steps (Fig. 5A), namely sirohydrochlorin formation and the incorporation of Fe^2+^ [78]. Strains unable to synthesize siroheme lack sulfite reductase activity due to deficiencies in Met10p and Met5p, resulting in methionine auxotrophy [53]. However, incorporating siroheme in the cofactor pseudoreaction did not alleviate the issue, as the growth of *MET1* and *MET8* mutants cannot be recovered when supplementing methionine. More sophisticated models, such as CofactorYeast [80], hold promise for better capturing and simulating these complex dependencies, thereby advancing the ability to decipher and predict metabolic intricacies.

The second scenario could be illustrated by spermine and spermidine (Fig. 5B). Polyamine biosynthesis contains multiple pathways, with the predominant route initiating via the transformation of ornithine into putrescine catalyzed by ornithine decarboxylase. Subsequently, putrescine is converted to spermidine by spermidine synthase, and spermidine is converted into spermine by spermine synthase [69,81]. Spermine can be converted to spermidine and β-alanine [82]. The interconversion between spermine and spermidine formed a closed loop, which inadvertently led to a zero flux prediction by the model for the spermidine synthesis pathway mediated by *SPE1* and *SPE3*. Based on the observation, we proposed that ignoring the growth-associated dilution of intracellular metabolites could result in biologically unrealistic flux distributions, thereby leading to erroneous predictions of gene essentiality, and thus algorithms that consider the dilution of intracellular metabolites such as MD-FBA [83] could be considered.

## 4 Conclusion

In this study, we proposed the auxotrophy-based method to curate GEMs and demonstrated its utility with the yeast consensus GEM Yeast9, improving the accuracy of the model in predicting gene essentiality and auxotrophic phenotypes and thereby enhancing its reliability and usefulness. Notably, the modifications can be directly implemented within the simulations for specific conditions e.g. growth on glucose, but the integration into the consensus model needs comprehensive examinations.

Our approach does not only involve the prediction of essential genes, a widely used curation approach, but also simulates the supplementation of corresponding compounds following the knockout of essential genes. This allows for examining both the pathways related to the essential genes and the pathways related to the utilization of the compounds, and thus serves as a more comprehensive curation manner. While we employed this approach in the yeast case, we foresee the potential applications of the method for curating GEMs of other organisms with available data. Additionally, the curated database of yeast auxotrophy stands as a resource for further research and model curation endeavors.

The continuous refinement of microbial cell factories has led us to leverage yeast consortia for executing more intricate tasks, minimizing the accumulation of intermediate metabolites while enhancing the synthesis of final products [84,85]. When transforming microbial cell consortia, it is common to block specific pathways in strains to establish symbiotic relationships among them. In co-cultivation scenarios, strains interrupt the synthesis of a certain essential compound by knocking out genes, relying on the production and supply of this compound by another strain in the consortium for growth. This aligns perfectly with the principle behind our curated model. In other words, the curated GEM would exhibit higher precision in the co-cultivation of auxotrophic strains. Employing the curated model, we systematically investigated the nutrient deficiency phenotypes of all genes, providing a valuable reference for the construction of nutrient-dependent yeast cell factories and consortia.

## Code availability

The code files are available for access at https://github.com/ChenYuGroup/AuxoYeast. The corrections are summarized and implemented in the function “auxoCurate”.

## CRediT authorship contribution statement

**Siyu Han:** Data curation, Formal analysis, Investigation, Software, Writing – original draft, Writing – review & editing. **Ke Wu:** Data curation, Formal analysis, Investigation, Writing – review & editing. **Yonghong Wang:** Supervision, Writing – review & editing. **Feiran Li:** Resources, Supervision, Writing – review & editing. **Yu Chen:** Conceptualization, Funding acquisition, Project administration, Resources, Supervision, Writing – original draft, Writing – review & editing.

## Declaration of competing interest

The authors declare that they have no known competing financial interests or personal relationships that could have appeared to influence the work reported in this paper.
