# Supplementary material for: Auxotrophy-based curation improves the consensus genome-scale metabolic model of yeast
Source: Synth Syst Biotechnol. 2024 Jul 30;9(4):861–70. doi: 10.1016/j.synbio.2024.07.006 (PMC11704421; doi:10.1016/j.synbio.2024.07.006)
Supplement: Supplementary information [file mmc1.pdf]

Supplementary information

Auxotrophy-based curation improves the consensus genome-scale metabolic model of yeast

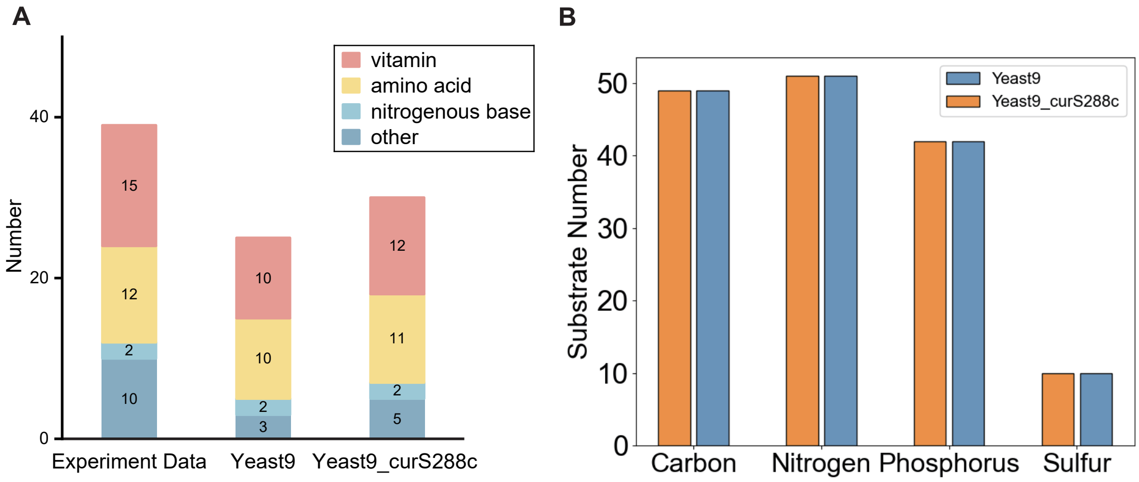

Figure S1. Performance of the GEM curated using data from S. cerevisiae S288c. (A) Number and classification of gene-compound corrections. (B) Simulations of substrate usage.

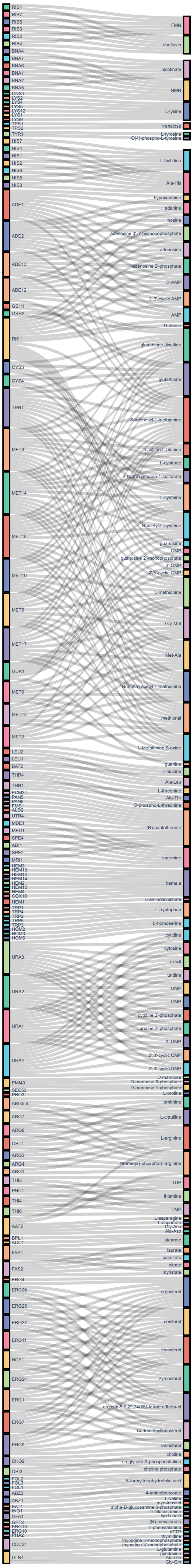
RIB1
RIB7
RIB5
RIB3
RIB2
RIB4
BNA4
BNA7
BNA6
BNA1
BNA2
BNA5
QNS1
LYS2
LYS4
LYS5
LYS12
LYS1
LYS9
TPS1
TPS2
TYR1
HIS7
HIS4
HIS1
HIS2
HIS6
HIS5
HIS3
ADE1
ADE2
ADE13
ADE12
GSH1
GSH2
RKI1
CYS3
CYS4
TRR1
MET3
MET14
MET16
MET10
MET5
MET17
GUA1
MET6
MET13
MET2
LEU2
LEU1
BAT2
THR4
THR1
ECM31
PAN5
PAN6
FMS1
ALD2
UTR4
MDE1
MEU1
SPE4
ADI1
SPE2
MRI1
HEM3
HEM13
HEM12
HEM14
HEM2
HEM15
HEM4
COX10
HEM1
TRP1
TRP4
TRP2
TRP5
TRP3
HOM2
HOM3
HOM6
URA3
URA2
URA1
URA4
PMI40
SEC53
PRO3
ARG5,6
ARG7
ARG8
ORT1
ARG3
ARG4
ARG1
THI5
PNC1
THI4
THI6
AAT2
BPL1
ACC1
FAS1
FAS2
ERG4
ERG26
ERG25
ERG27
ERG11
NCP1
ERG24
ERG1
ERG7
ERG9
CHO2
OPI3
FOL2
FOL3
FOL1
ABZ2
ABZ1
BAT1
INO1
GFA1
GPT2
ERG13
ERG10
PHA2
CDC21
GLN1
FMN
riboflavin
nicotinate
NMN
L-lysine
trehalose
L-tyrosine
O(4)-phospho-L-tyrosine
L-histidine
Ala-His
hypoxanthine
adenine
inosine
adenosine 3',5'-bismonophosphate
adenosine
adenosine 2'-phosphate
3'-AMP
2',3'-cyclic AMP
AMP
D-ribose
glutathione disulfide
glutathione
S-adenosyl-L-methionine
3-sulfino-L-alanine
L-cysteate
2-hydroxyethane-1-sulfonate
L-cysteine
N-acetyl-L-cysteine
guanosine
GMP
guanosine 2'-monophosphate
3'-GMP
2',3'-cyclic GMP
L-methionine
Gly-Met
Met-Ala
N(alpha)-acetyl-L-methionine
methional
L-Methionine S-oxide
guanine
L-leucine
Ala-Leu
L-threonine
Ala-Thr
O-phospho-L-threonine
(R)-pantothenate
spermine
heme a
5-aminolevulinate
L-tryptophan
L-homoserine
cytidine
cytosine
uracil
uridine
UMP
CMP
cytidine 2'-phosphate
uridine 2'-phosphate
3'-UMP
2',3'-cyclic CMP
2',3'-cyclic UMP
D-mannose
D-mannose 6-phosphate
D-mannose 1-phosphate
L-proline
ornithine
L-citrulline
L-arginine
N(omega)-phospho-L-arginine
TDP
thiamine
TMP
L-asparagine
L-aspartate
Gly-Asp
Ala-Asp
stearate
laurate
palmitate
oleate
myristate
ergosterol
episterol
fecosterol
zymosterol
ergosta-5,7,22,24(28)-tetraen-3beta-ol
14-demethyllanosterol
lanosterol
choline
sn-glycero-3-phosphocholine
choline phosphate
5-formyltetrahydrofolic acid
4-aminobenzoate
L-valine
myo-inositol
alpha-D-glucosamine 6-phosphate
D-Glucosamine
lipid chain
(R)-mevalonate
L-phenylalanine
dTTP
thymidine
thymidine 5'-monophosphate
thymidine 3'-monophosphate
L-glutamine
pyridoxine
Ala-Gln
Gly-Gln

Figure S2. Prediction of essential genes and their corresponding auxotrophs related to all metabolites. The genes represent the deficient genes in auxotrophic strains, and the compounds connected to them denote the nutrients required for these strains.
